# *Arabidopsis thaliana* ambient temperature responsive lncRNAs

**DOI:** 10.1186/s12870-018-1362-x

**Published:** 2018-07-13

**Authors:** Edouard Severing, Luigi Faino, Suraj Jamge, Marco Busscher, Yang Kuijer-Zhang, Francesca Bellinazzo, Jacqueline Busscher-Lange, Virginia Fernández, Gerco C. Angenent, Richard G. H. Immink, Alice Pajoro

**Affiliations:** 1 Max Planck Institute for Plant Breeding Research, 50829 Köln, Germany; 2 Laboratory of Phytopathology, Wageningen University and Research, 6708PB Wageningen, The Netherlands; 3 Laboratory of Molecular Biology, Wageningen University and Research, 6708PB, Wageningen, The Netherlands; 4 Bioscience, Wageningen University and Research, 6708PB Wageningen, The Netherlands

**Keywords:** Long non-coding RNA (lncRNA), Ambient temperature response, Flowering time, *Arabidopsis thaliana*, *FLINC*

## Abstract

**Background:**

Long non-coding RNAs (lncRNAs) have emerged as new class of regulatory molecules in animals where they regulate gene expression at transcriptional and post-transcriptional level. Recent studies also identified lncRNAs in plant genomes, revealing a new level of transcriptional complexity in plants. Thousands of lncRNAs have been predicted in the *Arabidopsis thaliana* genome, but only a few have been studied in depth.

**Results:**

Here we report the identification of Arabidopsis lncRNAs that are expressed during the vegetative stage of development in either the shoot apical meristem or in leaves. We found that hundreds of lncRNAs are expressed in these tissues, of which 50 show differential expression upon an increase in ambient temperature. One of these lncRNAs, FLINC, is down-regulated at higher ambient temperature and affects ambient temperature-mediated flowering in Arabidopsis.

**Conclusion:**

A number of ambient temperature responsive lncRNAs were identified with potential roles in the regulation of temperature-dependent developmental changes, such as the transition from the vegetative to the reproductive (flowering) phase. The challenge for the future is to characterize the biological function and molecular mode of action of the large number of ambient temperature-regulated lncRNAs that have been identified in this study.

**Electronic supplementary material:**

The online version of this article (10.1186/s12870-018-1362-x) contains supplementary material, which is available to authorized users.

## Background

LncRNAs represent a new class of recently discovered regulatory molecules. A key role for lncRNAs in the transcriptional and post-transcriptional regulation of a plethora of biological processes is emerging. LncRNAs are defined as long RNA molecules (longer than 200 bp) that do not code for a protein. They have been divided in different categories based on their location in the genome relative to protein-coding genes: natural antisense transcripts (NATs) are lncRNAs transcribed on the opposite strand of a protein-coding gene; long intergenic RNAs (lincRNAs) are transcribed in intergenic regions; intronic lncRNAs (iLncRNA) are transcribed from an intron in a protein-coding gene and; promoter lncRNAs (pLncRNAs), are transcribed from the promoter region of a gene [1, 2].

In the past years, genome-wide studies have identified many lncRNAs in the *Arabidopsis thaliana* (Arabidopsis) genome, as well as in other plant species, including rice and maize [1]; however the biological function of most of these lncRNAs is not known [3]. Currently, only a small number of lncRNAs has been functionally characterized and the emerging picture shows that lncRNAs have several modes of action, ranging from transcriptional interference to chromatin looping and mRNA splicing [1, 2, 4]. Moreover, these lncRNAs act in a wide range of biological processes. Some examples include the lincRNA APOLO, which is involved in auxin response [5], ELENA1, which functions in plant immunity [6], Enod40, which plays a role in regulation of sucrose utilization in nodules [7] and *IPS1, which* acts as a mimic of a miRNA target in the control of Pi homeostasis [8].

Vernalization, a long period of cold that is required by many temperate plant species to make the transition from the vegetative to the reproductive phase, is regulated by lncRNAs. The first characterized lncRNAs in plant were the lncRNAs transcribed from the *FLOWERING LOCUS C* (*FLC*) locus: the NAT COOLAIR [9], the iLncRNA COLDAIR [10], and more recently the pLncRNA COLDWRAP [11] and NAT ASL [12]. These molecules regulate the epigenetic silencing of *FLC* during vernalization in response to winter cold. This, together with the fact that the key regulators of flowering time in the ambient temperature pathway, *FLOWERING LOCUS M (FLM)* [13, 14], *MADS AFFECTING FLOWERING 2 (MAF2)* [15], *MAF3, MAF4* and *MAF5* [16], all belong to the *FLC* clade [17], prompted us to speculate a possible role for lncRNAs in ambient temperature-mediated development.

How plants are able to respond in a fast and dynamic manner to continuous changes in ambient temperature is a long-standing question in plant biology research. It is also important, in light of global warming, to understand how temperature affects plant development, as temperature influences different aspects of plant biology and development, including hypocotyl elongation, leaf shape determination, flowering time control and pathogen responses [18, 19]. Current research on the role of ambient temperature in plant development is mainly focused on identification and characterization of protein coding genes involved in this process. For example, MADS-box transcription factor genes, the bHLH transcription factor *PHYTOCHROME INTERACTING FACTOR 4 (PIF4)* [20–23], the *TEMPRANILLO* genes [24] and the MYB transcription factor *EARLY FLOWERING MYB PROTEIN (EFM)* [25] have been shown to regulate ambient temperature-mediated flowering. PIF4, together with the photoreceptor phytochrome B (phyB), also plays an important role in thermo-morphogenesis [26–28]. Furthermore, there is an important role in ambient temperature-mediated responses for various circadian clock genes [29–31].

Despite the clear role for lncRNAs in cold temperature signaling, their potential role as mediators of ambient temperature responses remains unexplored.

## Results

### Identification of lncRNAs in Arabidopsis thaliana.

We used RNA-seq transcriptome analysis to identify lncRNAs in shoot apical meristem (SAM)-enriched tissue that might play a role in ambient temperature-mediated developmental processes. We focused on transcripts carrying a polyA tail and followed a TAIR10-guided assembly approach using cufflinks. This approach allowed the identification of both known lncRNAs and annotation of novel transcripts [32]. Strand-specific libraries were generated from shoot apical meristem (SAM)-enriched tissue of 5 weeks-old Arabidopsis Col-0 plants grown in short-day conditions, and therefore still in the vegetative stage. The SAM of plants grown in short-day conditions is larger than the SAM of those grown in long-day conditions [33], allowing the presence of a good proportion of SAM tissue in the sampled material. Using this approach we aimed to identify low-expressed transcripts in the SAM. To annotate the transcripts, the “cuffcompare” function of cufflinks was implemented, which classifies transcripts base on their relationship with the TAIR10 reference genome annotation. A similar approach was recently used successfully for the identification of lncRNAs in the tomato genome [34].

We selected transcripts that belong to the class code “x”, “i” and “u” (Table 1). The class code “x” defines “Exonic overlap with reference on the opposite strand”, and includes, among others, the Natural Antisense Transcripts (NAT). The class code “i” defines “transcribed genomic fragments falling entirely within a reference intron”, which includes the intronic lncRNAs (iLncRNA). Finally, the class code “u” defines the “unknown intergenic transcript”, e.g. the long intergenic lncRNA (LincRNAs) and the promoter lncRNAs (pLncRNAs). In total, we identified 2132 new putative lncRNA that belong to one of these categories (Table 1 and Additional file 1: Table S1).

**Table 1:** putative lncRNAs

*Category*	*abbreviation*	*cufflink class*	*number*	
Natural antisense transcript	NAT	x	367	
long intergenic ncRNA	LincRNA	u	1591	
intronic lncRNA	iLncRNA	i	174	
			2132	total

Only a few lncRNAs are annotated in the TAIR10 annotation. Therefore, we retrieved an extensive list of 14,954 RNAs annotated as lncRNAs from the Plant long non-coding RNA database (PlncDB) [35] and compared these with our identified putative lncRNA transcripts. In total 644 of the 2132 lncRNA identified in our annotation were also annotated in the PlncDB, 22 iLncRNA, 92 NATs and 530 lincRNAs (Additional file 1: Table S1).

Next, we compared the lncRNAs identified in our analysis with the ones reported in the CANTATAdb 2.0 [36]. We found 321 transcripts to be also present ion the CANTATA database, 10 iLncRNA, 38 NATs and 273 lincRNAs (Additional file 1: Table S1).

A new annotation of the Arabidopsis genome, Araport11, was released recently [37]. This new annotation is based on the analysis of a collection of RNA-seq experiments performed on different tissues from plants grown under different conditions. This approach allows us to identify new lncRNA transcripts expressed in specific conditions. We investigated the overlap between our lncRNA annotation and Araport11 transcripts and found 457 lncRNAs in both data sets (Additional file 1: Table S1).

Remarkably, 1323 lncRNAs were uniquely identified in our analysis, highlighting the power of our approach in isolating potentially new lncRNAs. Only a small number of these are likely to be falsely assigned due to sequencing or annotation errors. The identification of this relatively large number of novel transcripts is likely due to the fact that lncRNA are generally lowly expressed and therefore, their identification is dependent on the experimental setup used and tissues sampled for the RNA isolation.

Because one of the key developmental processes regulated by ambient temperature is flowering time control, we initially compared the genomic location of the lncRNAs in relation to the localization of key flowering time genes. A thorough investigation revealed that 22 lncRNAs are located nearby a flowering time gene [38], such as the lincRNA *AtLnc1134* located in *ABRE BINDING FACTOR 4* (*ABF4*), the *AtLnc1860*, *AtLnc198* and *AtLnc236*, located respectively in the promoters of *MADS AFFECTING FLOWERING 2* [15, 39, 40], *FLAVIN-BINDING, KELCH REPEAT, F Box 1*(*FKF1*) [41, 42] and *EARLY FLOWERING IN SHORT DAYS* (*EFS*) [43] (Additional file 2: Table S2).

### LncRNA expression is modulated by ambient temperature

**Fig. 1:**
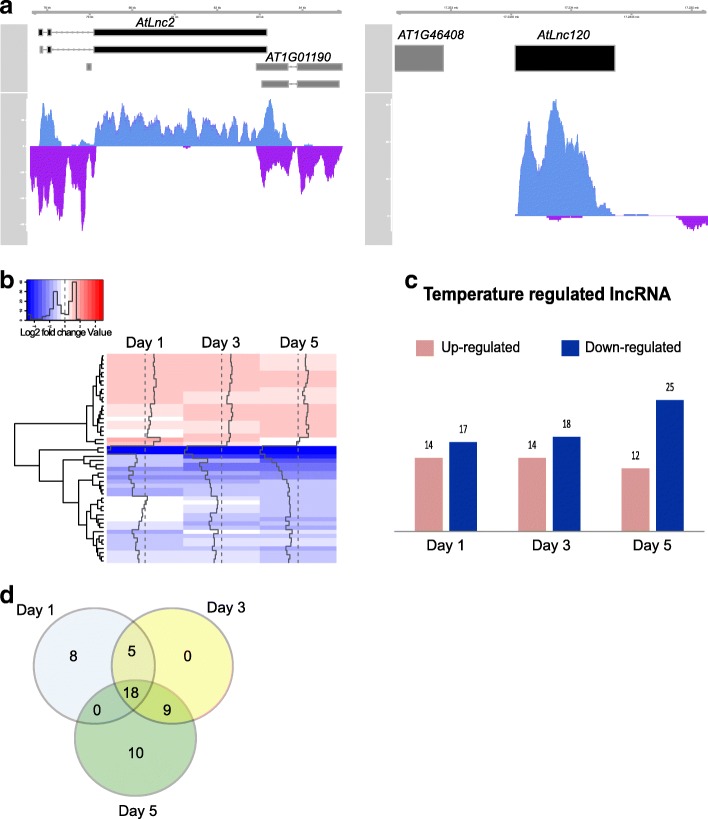
Identification of ambient temperature-responsive lncRNAs. **a**. Example of a lncRNA identified in our strand-specific RNA-seq experiment. Protein coding genes and lncRNA are shown in grey and black respectively. In blue are reads mapping to the forward strand and in purple are reads mapping to the reverse strand from RNA-seq on plants growing at 16 °C in short day. **b**. Heat map showing lncRNA that are significantly (*adj* *p*-value < 0.01) differentially expressed (log2 fold change > |1|) in at least in one time point upon a temperature change from 16 °C to 25 °C. **c**. Histogram showing the number of lncRNAs whose expression is up-regulated and down-regulated at each time point after the temperature change. **d**. Venn diagram showing the overlap in differentially expressed lncRNA at each time point

To identify lncRNAs that are responsive to an ambient temperature change, we compared the transcriptome of plants that were grown in short day condition at 16 °C and then moved to 25 °C with the transcriptome of plants grown continuously at 16 °C. For this purpose, tissue was collected at one, three and 5 days after the temperature increase, as described previously [44]. When grown in short-day conditions meristem size is increased, providing a relative higher amount of meristematic tissue to study the effect of a change in ambient growth temperature on the expression of meristematic lncRNAs. Using this approach, we found 50 lncRNAs (11 NATs, two intronic lncRNA and 37 lincRNAs) that were significantly differentially- expressed (log_2_ fold change> |1|, *p*-value < 0.01) in at least one time point upon the temperature change (Additional file 3: Table S3). Among the temperature responsive lncRNAs are *AtLnc2* (Fig. 1a), an antisense RNA of *CYTOCHROME P450 FAMILY 78, SUBFAMILY A, POLYPEPTIDE 8 (CYP78A8A),* which plays a role in reproduction [45], *AtLnc120* a LincRNA located nearby the MADS-box transcription factor *AGL97* (Fig. 1a**)**, and *AtLnc1128* located in the intron of *HOMOLOGUE OF CYANOBACTERIAL RBCX 1 (RBCX1)*, coding for a RUBISCO chaperon involved in cold response [46, 47]. We observed that expression of 28 of the differentially expressed lncRNAs (DElnc) was repressed upon an increase in growth temperature, while 22 lncRNAs showed an increase in expression (Fig. 1b-c). The majority of DElnc (36%) are differentially expressed at all time-points, while 16% of the DElnc are only differentially expressed 1 day after the temperature switch, showing a rapid and transient temperature response. 20% of the DElnc appeared to be differentially expressed at day five only, showing a late and probably indirect response to the temperature change (Fig. 1d).

Subsequently, co-regulation was investigated for NATs and lincRNAs (Additional file 4: Table S4). In the case of NATs we compared their expression level to the expression of their corresponding sense transcript while for the lincRNAs to their two direct flanking protein coding genes. In general, we observed a positive Pearson correlation between sense and antisense transcript and we found the expression of the sense transcript was either not changed or changed in the same direction as the NAT (Additional file 4: Table S4). We also observed correlation (with prevalence of positive correlation) for most of the lincRNAs with at least one of the flanking genes, suggesting co-regulation between the lncRNAs and the protein coding genes located nearby (Additional file 4: Table S4). For example, *AtLnc1444* showed a positive correlation with both the two direct flanking protein coding genes, *C-REPEAT/DRE BINDING FACTOR 1* (*CBF1*) and *CBF3*, genes involved in response to low temperature [48–51], while *AtLnc488* showed a negative correlation with the transcription factor *TEOSINTE BRANCHED1/CYCLOIDEA/PCF 15* (*TCP15*) [52, 53].

### AtLnc428 plays a role in temperature-mediated flowering

We selected five ambient temperature responsive lncRNA for further functional characterization. We focused on lncRNAs for which a T-DNA insertion line is available in the predicted transcript or promoter region. We hypothesized that the promoter T-DNA insertions will affect the lncRNA expression level or pattern. We investigated the role of the temperature responsive *AtLnc2*, *AtLnc120*, *AtLnc213*, *AtLnc428* and *AtLnc1524* in the regulation of temperature-mediated flowering. Expression of *AtLnc2* and *AtLnc120* is up-regulated upon temperature increase while expression of *AtLnc213*, *AtLnc428* and *AtLnc1524* is down-regulated (Additional file 3: Table S3).

**Fig. 2:**
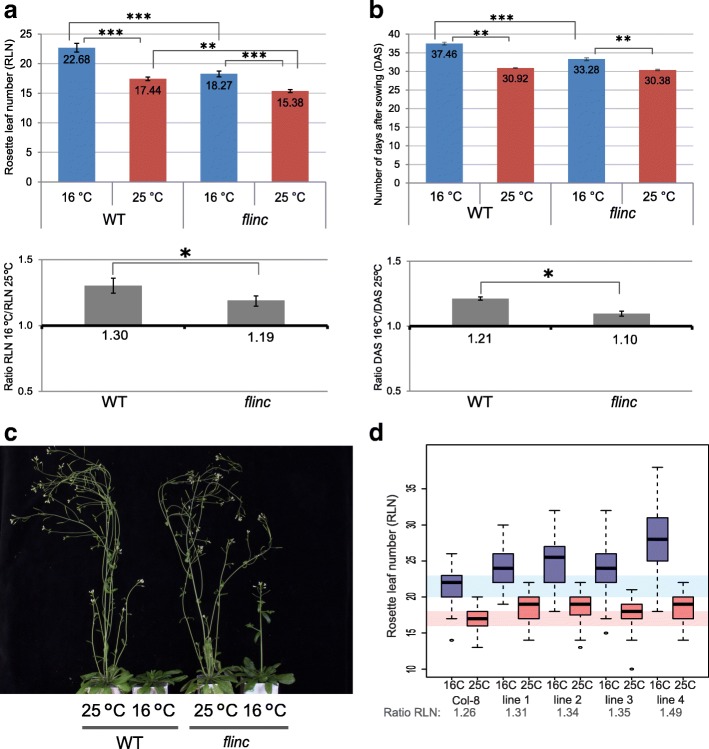
*FLINC* plays a role in temperature-mediated flowering. **a**. Flowering time measured as rosette leaf number (RLN) for *FLINC* wild-type and mutant plants growing at 16 °C and 25 °C in long day conditions. The experiment was performed using four biological replicates with 13 plants per replicate for each genotype/condition. **b**. Flowering time measured as days after sowing (DAS) for *FLINC* wild-type (WT) and mutant plants growing at 16 °C and 25 °C in long day conditions. The experiment was performed using four biological replicates with 13 plants per replicate for each genotype/condition. **c**. Eight weeks-old *FLINC* WT and mutant plants grown at 16 °C and 25 °C in long day conditions. **d**. Flowering time in RLN for four independent T2 lines overexpressing FLINC (FLINC-OE) at 16 °C (blue) or 25 °C (red) in long day conditions. Box-plot showing the distribution of ca. 40 plants per replicate for each genotype/condition. FLINC-OE plants are more sensitive to temperature than the WT as shown by the higher RLN ratio. *** indicates significant differences at *p*-value < 0.0001, ** indicates significant differences at *p*-value < 0.001, and * indicates significant differences at *p*-value < 0.05 according to the Student t-test

Wild-type and T-DNA insertion plants were grown for 3 weeks at 16 °C in long day conditions, and were phenotyped for their flowering time response after the switch in growth temperature to 25 °C and in comparison to plants that were maintained at 16 °C. Flowering time was quantified by counting rosette leaf number (RLN) and the number of days from sowing to the appearance of the main inflorescence (days after sowing; DAS). We measured the effect of temperature on flowering time as the ratio of RLN or DAS between mutant and Col-0 plants growing at 16 °C and 25 °C. Genotypes with complete temperature insensitivity will show a ratio of 1 [54]. Among the five lines tested, only the line carrying a T-DNA insertion in the *AtLnc428* locus showed a significant change in temperature-mediated flowering compared to the wild-type (Additional file 5: Figure S1 and Fig. 2). We found *AtLnc428* mutant plants to be significantly less sensitive to temperature-mediated flowering (Fig. 2a-c) and, for this reason, renamed this lncRNA ‘*FLOWERING LONG INTERGENIC NON CODING RNA* (*FLINC*)’.

*FLINC* is an intergenic lncRNA located in chromosome 1 between *At1g56233*, a gene coding for a defensin-like (DEFL) family protein and *At1g56240*, a gene coding for a phloem protein 2-B13. *At1g56233* and *At1g56240* are located respectively at 1.5 kb and 3.2 kb from *FLINC* (Additional file 6: Figure S2A). The effect of T-DNA insertion in *FLINC* on expression of *FLINC* was examined by qPCR by comparing wild-type and mutant plants in a segregating population (Additional file 6: Figure S2B). No *FLINC* expression was observed in plants with the T-DNA insertion, suggesting that the *flinc* mutant is a full knock-out. We also examined the effect of the *FLINC* T-DNA insertion on the expression of the genes flanking *FLINC* in all above ground tissues of 2 weeks-old plants in the vegetative stage of development. *At1g56233* expression could not be detected in either wild type Col-0 or *flinc* plants, and we did not observe any significant change in the expression of *At1g56240* (Additional file 6: Figure S2C). This result indicates that the T-DNA insertion does not affect the expression of the genes flanking *FLINC* in the investigated material*.*

Our results indicate a role for *FLINC* in the regulation of ambient temperature-mediated flowering. To confirm this observation, we generated plants overexpressing the lncRNA under control of the constitutive CaMV35S promoter and analyzed their flowering behavior at different ambient temperature conditions (Fig. 2d). In contrast to the *flinc* T-DNA insertion line, we found that plants overexpressing the lncRNA transcript are more sensitive to a temperature change than wild-type plants, and providing additional evidence for the role of *FLINC* in the control of ambient temperature-mediated flowering.

In our RNA-seq experiment, *FLINC* was down-regulated upon the temperature change under short day conditions (Additional file 3: Table S3). Because flowering time investigations were performed under long day conditions, we examined the effect of an increased ambient temperature on *FLINC* expression under these conditions. This analysis revealed a similar response upon an increase of temperature from 16 °C to 25 °C as was observed in the initial experiment under short day conditions (Additional file 6: Figure S2E), suggesting that regulation of *FLINC* expression by temperature is not day-length dependent.

Since a number of genes involved in the ambient temperature pathway are circadian-regulated or code for circadian clock components [55], we monitored the expression of *FLINC* in a 24 h-time course. We found that *FLINC* expression is not influenced by the circadian rhythm (Additional file 6: Figure S2F).

We also examined the *FLINC* expression profile during Arabidopsis development and in different Arabidopsis tissues (Additional file 6: Figure S2G and Figure S2H). We found that *FLINC* is expressed broadly in the plant, suggesting that *FLINC* plays a more extensive role during development than just the control of flowering.

To shed some light on the molecular basis of the *flinc* phenotype, we investigated the expression of selected flowering-related genes in wild type and 3 weeks-old *flinc* plants grown in long day conditions at 16 °C. These include the flowering repressors *FLC* and *FLM*, the florigen *FLOWERING LOCUS T* (*FT*) and the floral integrators *APETALA 1* (*AP1*), *SUPRESSOR OF COSTANS 1* (*SOC1*), *LEAFY* (*LFY*) and *TERMINAL FLOWER 1 (TFL1)* (Additional file 7: Figure S3A). We detected higher expression of *FT* in *flinc* than in the Col-0 wild type, in agreement with the early flowering phenotype of *flinc* (Fig. 2a).

LncRNAs have been show to exert their effect in numerous ways, including nucleotide pairing with mRNAs [1] of protein-coding genes or miRNAs. Therefore, we searched for regions homologous to *FLINC* within the Arabidopsis genome. We found ten regions with partial sequence homology to *FLINC*, of which five are located within protein coding genes. However, for none of these five genes differential expression was found in *flinc* in comparison to Col-0 wild type (Additional file 8: Table S5).

To obtain insight in conservation of *FLINC*, we searched for *FLINC*-related lncRNAs in genomes of other plant species using Blast and found various similar genes (e-value < 0.001), e.g. in the close Arabidopsis relatives *Arabis alpina* and *Brassica rapa,* but also in less-related species such as *Solanum pennelli* and *Vitis vinifera* (Additional file 7: Figure S3C). These results point to positive selection on *FLINC.* Further research is needed to confirm the proposed role of *FLINC* in ambient temperature-mediated flowering time control in Arabidopsis and to explore the function of the closely related genes in other species.

## Discussion

The discovery of lncRNAs as regulatory molecules revealed a new layer of complexity in gene expression regulation. So far, only few of the thousands of identified lncRNAs in plants have been functionally characterized. Nevertheless, these studies revealed that lncRNAs play a role in many different biological processes, from lateral root development to photomorphogenesis [1, 2]. We identified new Arabidopsis lncRNAs in SAM-enriched tissue and show ambient temperature-sensitive expression for a small subset of these lncRNAs. Furthermore, we showed that one of these lncRNAs, *FLINC*, regulates temperature-mediated flowering in Arabidopsis. Previous studies showed that expression of the lncRNAs transcribed from the *FLC* locus, COOLAIR [9], COLDAIR [10], ASL [12] and COLDWRAP [11], is regulated by a prolonged period of cold (4 °C). By contrast, *FLINC* expression is affected rapidly by a milder change of temperature. *FLINC* is an intergenic lncRNA and at this moment it is not clear which genes are regulated by *FLINC*. Thus, further research is needed to elucidate *FLINC’s* molecular mode of action. The observation that *FT* is higher expressed at low temperature in *flinc* mutant plants, suggests that *FLINC* acts prior to the integration of the environmental signals by FT. Next, the two transcription factors with a known role in temperature-mediated flowering time control, SOC1 [56] and AGAMOUS-LIKE 15 (AGL15) [57], bind to the *FLINC* locus suggesting a regulatory interaction. Moreover, the presence of sequence conservation in different plant species points to positive selection and a potential conserved role for *FLINC* in ambient temperature-mediated flowering time control.

Advances in RNA sequencing technology allow a more precise identification of lncRNA transcripts. Thanks to the use of strand specific RNA-seq data, it is now possible to identify transcripts derived from the opposite stand and therefore, get insight into expression profiles of NATs. The current sequence depth that can be obtained facilitates identification of low abundant transcripts, including as numerous lncRNAs. LncRNA expression appeared to be heavily responsive to environmental conditions, including temperature (this study) or drought [58], suggesting a role for these molecules in plant adaptation to the environment. The tissue-specific and environmental regulation of lncRNAs illustrates the need for defined experiments to identify all lncRNA-encoding genes in the genome.

## Conclusions

The discovery of lncRNA as regulatory molecules revealed a new layer of complexity in the regulation of gene expression, and functional studies in plants revealed roles for lncRNAs in various biological processes. Here we show that lncRNA expression is influenced by ambient temperature changes and identified a significant role in ambient temperature-mediated flowering time the *FLINC* lncRNA. The challenge for the future is to decipher the biological function and molecular mode of action of ambient temperature responsive lncRNAs.

## Methods

### RNA-seq experiments

RNA-seq data were generated previously by Pajoro et al., 2017 [44]. In brief, plants were grown under short day conditions (8 h light, 16 h dark) on rock-wool in growth cabinets with LED lamps with light intensity of 200 μmol m-2 s-1 and 75% relative humidity at 16 °C for 5 weeks. Nutrients were supplied by sub-irrigation with Hyponex. After 5 weeks, plants were either left at the same temperature or moved to 25 °C (6 h after lights on). Three biological samples were generated for each condition. For each sample, tissue from ca. 10 plants was collected 4 hours after light on. Using jeweler’s forceps, leaves were removed to obtain SAM-enriched tissue. Total RNA was extracted using the Invitek InviTrap Spin Plant RNA Mini Kit (REF: 1064100300) according to the manufacturer’s protocol. DNase treatment was performed to remove genomic DNA. DNase I digestion was performed on total RNA using Turbo DNase from Ambion according to the manufacturer’s protocol. RNA integrity was checked on a by agarose gel electrophoresis after DNase I treatment. Samples were prepared for Illumina sequencing using the Illumina TruSeq Stranded mRNA Sample Prep kit (REF: 15032613) according to the manufacturer’s protocol, which generates polyadenylated transcripts. Libraries were analyzed on the Bioanalyzer and quantified using a Qubit fluorometer before pooling for sequencing on an Illumina HiSeq2500. Two lanes were used on a 125 bp paired-end (PE) run.

### RNA-seq data analysis

RNA-seq reads were mapped against the Arabidopsis genome version TAIR10 (www.arabidopsis.org) using TopHat2 [59]. To identify un-annotated transcripts, reference based full length transcript reconstruction was performed separately for each sample using Cufflinks with strand-specific awareness [32]. Cuffmerge, which is part of the cufflinks package, was finally used for merging the individual cufflinks results into an overall set of full-length transcripts. The function “Cuffcompared” was used to compare the newly annotate transcripts with the TAIR10 reference annotation. We selected transcripts that belong to the class code “x”, “i” and “u”. The class code “x” defines “Exonic overlap with reference on the opposite strand”, to this class belong e.g. the Natural Antisense Transcripts (NAT). The class code “i” defines “transcribed genomic fragments falling entirely within a reference intron”, to this class belong the intronic lncRNAs (iLncRNA). Finally, the class code “u” defines the “unknown intergenic transcript”, to this class belong e.g. the long intergenic lncRNA (LincRNAs). To retrieve differentially expressed transcripts upon the temperature changes, the number of fragments mapping to the newly annotated transcripts was determined using HTseq count [60]. Differentially expressed genes were detected using DESeq2 [61].

### Flowering time assay

Seeds of T-DNA insertion lines (Table 2), were obtained from the Nottingham Arabidopsis Stock Center (NASC). Plants were genotyped using the primers listed in Table 2. The progeny of mutant and wild-type plants from the segregating population was used in the flowering time experiments for *AtLnc213* and *AtLnc428*. Since *AtLnc2, AtLnc120* and *AtLnc1524* homozygous T-DNA insertion lines were obtained from the Stock Center, Col-8 wild-type plants were used as comparison. For flowering time analyses, plants were randomly arranged in trays and grown in growth cabinets, as described above, but in long day-conditions (16 h light, 8 h dark). After 3 weeks of growth at 16 °C, half of the plants were moved to the 25 °C growth cabinet with the same day length, light intensity and humidity conditions. Flowering time was quantified by determining the time until the macroscopic appearance of the first flower bud (days after sowing, DAS; screening was done every day), and by counting rosette leaf numbers (RLN) per plant after bolting of all plants in the tray. Four biological replicates were used, with 13 plants per replicate for each genotype/condition. Data are shown as the average of ratios between the replicates. Student’s t-tests were calculated with GraphPad QuickCalcs.

**Table 2:** lncRNAs T-DNA insertion lines

lncRNA	T-DNA line	primer LP	primer RP
*AtLnc2*	SALK_006791	TACTCCATGCATTGATGCTTG	AAACACTGACTTGACGGCATC
*AtLnc120*	SALK_056929	AGCAGCGACGACATTATCAAC	CATCGTCTTCTTCTTCCGTTG
*AtLnc213*	SALK_009581	AGGAGGTTGAGAGCAAGGAAG	CGGTAACTGAATCAAAGCCAC
*AtLnc428*	SALK_025080	AACAATTAGGCAAGGTTTGGG	TTCATCATAGTCTCCATCGGG
*AtLnc1524*	SAIL_896_E02	TGAAGCGAACCTACATCTTGG	ACCTAGCATCGTAGGTAGGCG

### Expression profile by qRT-PCR

Plant were grown under long day conditions (16 h light, 8 h dark) at 21 °C as described above. Three biological samples were generated for each condition tested, unless stated otherwise. For each sample, tissue from ca. 10 plants was collected using jeweler’s forceps. Total RNA was extracted and DNAse-treated as described above. cDNA was synthetized using the iScript kit from BIORAD (Cat. No. 1708890) using one μg of total RNA. Gene expression was measured by quantitative RT-PCR (qPCR) using the iQ™ SYBR® Green Supermix from BIORAD (Cat. No. 1708885). The qPCR primers are listed in Additional file 9: Table S6. Relative expression was calculated using *TIP41* as reference gene for normalization and the formula 2^-(ΔCt^_target_^– ΔCt^_TIP41_^)^. Normalized fold expression was calculated by setting the wild-type (WT) value to one.

### Generation of FLINC overexpression lines

Gateway cloning was used to generate the *FLINC* overexpression construct. The FLINC transcript was amplified by PCR from cDNA and cloned into the pDONR201 entry vector using a BP reaction. The entry vector was then recombined with the destination vector pB7FWG2 carrying the CaMV35S promoter. The resulting expression vector with the *p35S:FLINC* construct was transformed into Arabidopsis Col-0 plants by floral dip. Transformed plants were selected on media with 10 μg/ml of phosphinothricin.

### Identification of protein coding genes with sequences homologous to *FLINC*

We used the BLASTN software [62] with default parameter to identify Arabidopsis protein coding genes with sequence similarity to *FLINC*. The genome coordinates that matched the *FLINC* sequence were compared to the coordinates of the Arabidopsis gene models using the Bedtools intersect software [63] and the gene coordinates as reference and –F 1 parameter. To identify sequences similar to *FLINC* in other plant species, we used Blastn software (NCBI) [64] with the parameter optimized for ‘somewhat similar sequences’. Only species with an e-value < 0.001 were considered.

## Additional files

Additional file 1: **TableS1**. LncRNAs annotation. List of lncRNAs retrieved in this study and corresponding identification in the Plant long non-coding RNA database (PlncDB) and Araport11. (XLSX 422 kb)

Additional file 2: **TableS2**. List of lncRNAs located within a 5 kb region of a gene involved in flowering time regulation. (XLSX 15 kb)

Additional file 3: **Table S3**. Differentially expressed lncRNAs (DElnc). List of lncRNAs differentially expressed between 16 °C and 25 °C day 1, 16 °C and 25 °C day 3, 16 °C and 25 °C day 5. DElnc are defined as transcripts with a change in expression of log2 Fold Change |1| and adjusted *p*-value according to the BH method for controlling false discovery < 0.05. (XLSX 14 kb)

Additional file 4: **Table S4**. Pearson correlation between lncRNAs and protein coding genes. For the NATs expression value of the corresponding sense transcript while for the lincRNAs the expression value of their two direct flanking protein coding genes in our data set was used. NA, indicates absence of detectable expression in our RNA-seq. (XLSX 15 kb)

Additional file 5: **Figure S1**. Temperature induced flowering for T-DNA insertion lines. Col-8 plants were used for comparison with AtLnc2 (A), AtLnc120 (B), and AtLnc1524 (D) T-DNA insertion lines, while wild-type and T-DNA carrying plants from segregating population were compared for AtLnc213 (C). The experiment was performed using four biological replicates with 13 plants per replicate for each genotype/condition. The T-DNA insertion did not affect temperature-induced flowering in any of these mutants since no significant difference was observed in the ratio of flowering time at the different temperatures between wild-type and mutant plants. (TIF 642 kb)

Additional file 6: **Figure S2**. A. FLINC location in the genome. B. FLINC expression in WT and flinc mutant plants. The graph shows the average of three biological replicates, each composed of a pool of 10 2 weeks-old plants. Plants were growing at 21 °C in long day conditions. Bars indicate SEM of the replicates. Plants with a T-DNA insertion in the lncRNA locus do not show detectable expression of the lncRNA transcript. C. At1g56233 expression in WT and flinc mutant plants. The graph shows the average of three biological replicates, each composed of a pool of 10 2 weeks-old plants. Plants were growing at 21 °C in long day conditions. Bars indicate SEM of the replicates. No significant difference in At1g56233 expression was observed in flinc, *p*-value equals 0.3439 according to the T-test. D. FLINC expression in WT and FLINC-OE plants. A pool of 10 2 weeks-old plants growing on selection medium at 21 °C in long day was used for the analysis. E. FLINC expression measured by qPCR in plants growing at 16 °C and 25 °C in long days. Expression is relative to the level at 16 °C. Bars indicate SEM of two biological replicates, each composed of a pool of seven plants. FLINC expression is significantly lower at 25 °C compared to 16 °C (p-value = 0.0467, Students’ t-test). F. FLINC expression during a 24 h time course in plants grown at 21 °C in long days. The graph shows the average of four biological replicates, each composed of a pool of 25 ten days-old plants. Plants were growing at 21 °C in long day conditions. Bars indicate SEM of the replicates. G. FLINC expression measured by qRT-PCR in different plant tissues. The graph shows the average of three biological replicates, each composed of a pool of 6 to 8 plants for all tissues, except for ‘siliques’ and ‘stems’, for which only two biological replicates were used. Bars indicate SEM between the replicates. Plants were growing at 21 °C in long day conditions. H. FLINC and AP1 expression measured by qPCR in rosettes during a development time course at 21 °C in long day conditions. The graph shows the average of three biological replicates, each composed of a pool of 6 to 8 plants. Bars indicate SEM between the replicates. (PDF 476 kb)

Additional file 7: **Figure S3**. A. Expression of flowering-related genes in WT and flinc plants. Expression was measured by qPCR in rosettes of twenty days-old WT and flinc grown at 16 °C in long day conditions. Material was harvested at ZT6. The graph shows the average of three biological replicates, each composed of a pool of 10 plants. Bars indicate SEM of the replicates. B. Expression of genes with sequence similarity to FLINC in wild-type (WT) and flinc mutant plants (Mut), as measured by qPCR. The graph shows the average of three biological replicates, each composed of a pool of 10 two weeks-old plants growing at 21 °C in long day. Bars indicate SEM of the replicates. C. FLINC sequences are also found in other plant species. (PDF 561 kb)

Additional file 8: **Table S5**. Arabidopsis thaliana genomic region with sequence homology to FLINC. (XLSX 11 kb)

Additional file 9: **Table S6**. List of primers used for qPCR. (XLSX 9 kb)

## Abbreviations

ABF4: ABRE BINDING FACTOR 4
AGL15: AGAMOUS-LIKE 15
AP1: APETALA1
CYP78A8A: CYTOCHROME P450 FAMILY 78, SUBFAMILY A, POLYPEPTIDE 8
DAS: days after sowing
EFM: EARLY FLOWERING MYB PROTEIN
EFS: EARLY FLOWERING IN SHORT DAYS
FKF1: FLAVIN-BINDING, KELCH REPEAT, F Box 1
FLC: FLOWERING LOCUS C
FLINC: FLOWERING LincRNA
FLM: FLOWERING LOCUS M
iLncRNA: intronic lncRNAs
LFY: LEAFY
lincRNAs: long intergenic RNAs
lncRNAs: Long non-coding RNAs
MAF: MADS AFFECTING FLOWERING
NATs: natural antisense transcripts
NGS: Next Generation Sequence.
PIF4: PHYTOCHROME INTERACTING FACTOR 4
PlncDB: Plant long non-coding RNA database
RBCX1: HOMOLOGUE OF CYANOBACTERIAL RBCX 1
RLN: rosette leaf number
SOC1: SUPPRESSOR OF OVEREXPRESSION OF CO 1
SVP: SHORT VEGETATIVE PHASE
TFL1: TERMINAL FLOWER 1
