# Supplementary figures and images for: Arabidopsis thaliana ambient temperature responsive lncRNAs
Source: BMC Plant Biol. 2018 Jul 13;18:145. doi: 10.1186/s12870-018-1362-x (PMC6045843; doi:10.1186/s12870-018-1362-x)

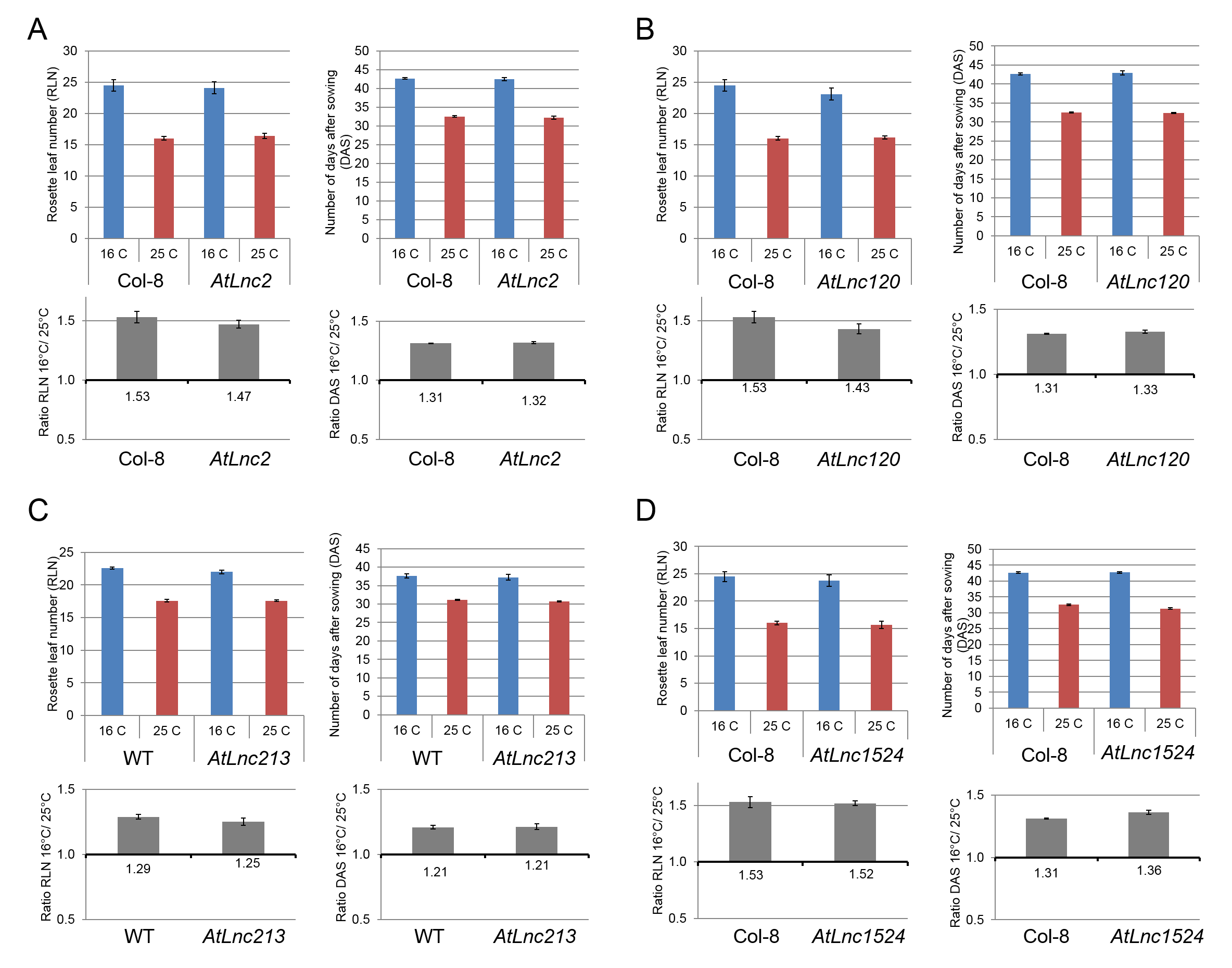

Supplement: Supplementary file 5 — Figure S1. Temperature induced flowering for T-DNA insertion lines. Col-8 plants were used for comparison with AtLnc2 (A), AtLnc120 (B), and AtLnc1524 (D) T-DNA insertion lines, while wild-type and T-DNA carrying plants from segregating population were compared for AtLnc213 (C). The experiment was performed using four biological replicates with 13 plants per replicate for each genotype/condition. The T-DNA insertion did not affect temperature-induced flowering in any of these mutants since no significant difference was observed in the ratio of flowering time at the different temperatures between wild-type and mutant plants. (TIF 642 kb) [file 12870_2018_1362_MOESM5_ESM.tif]

A

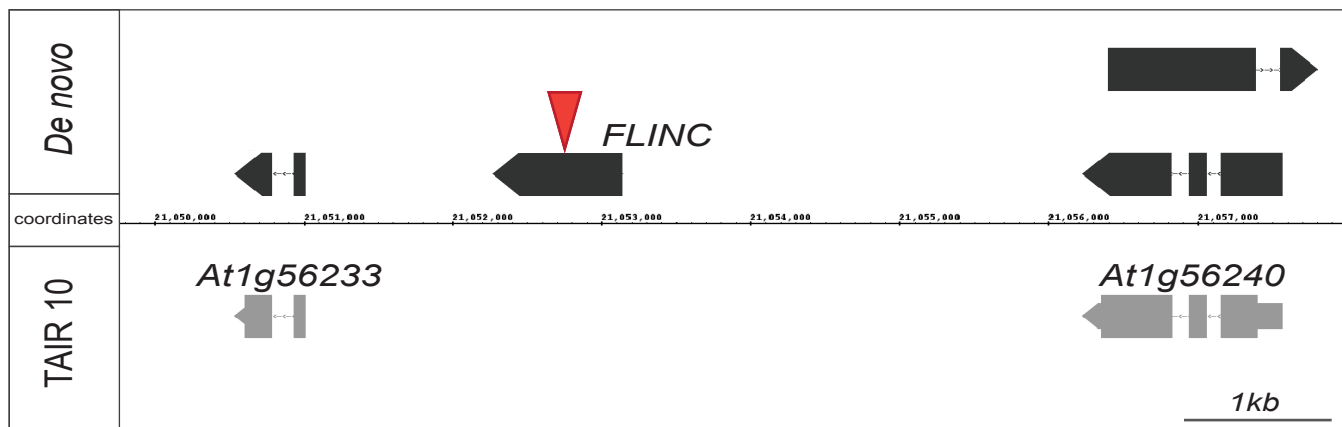

B

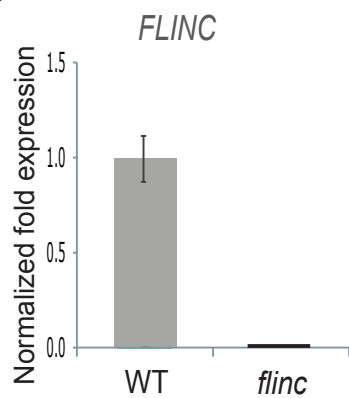

C

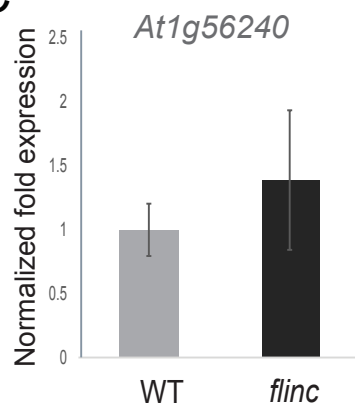

D

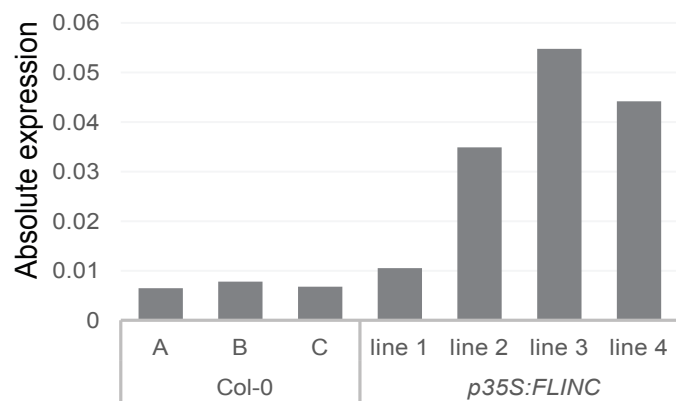

E

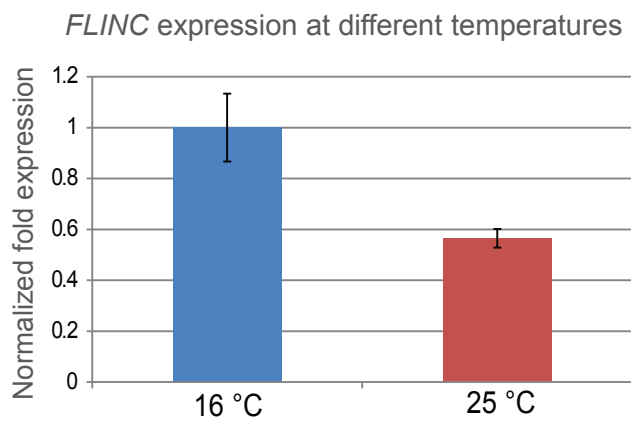

F

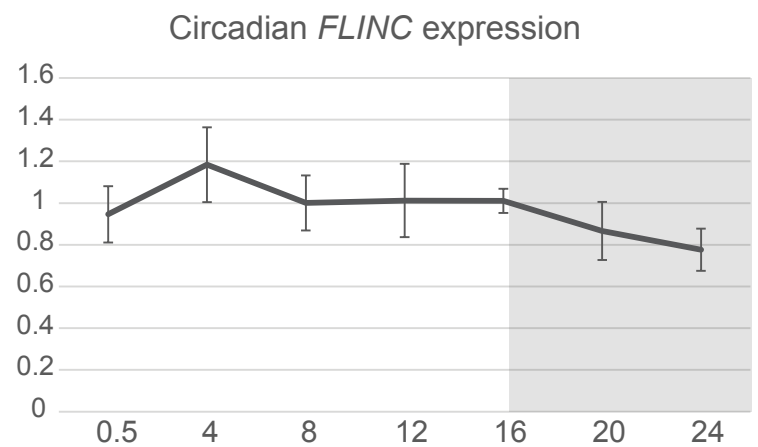

G

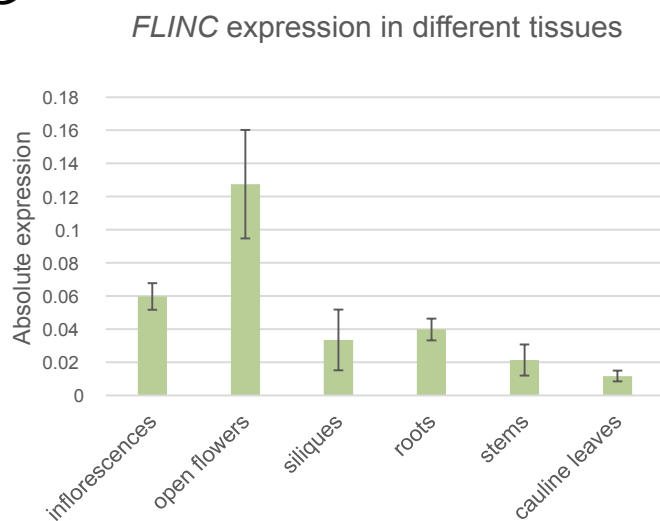

H

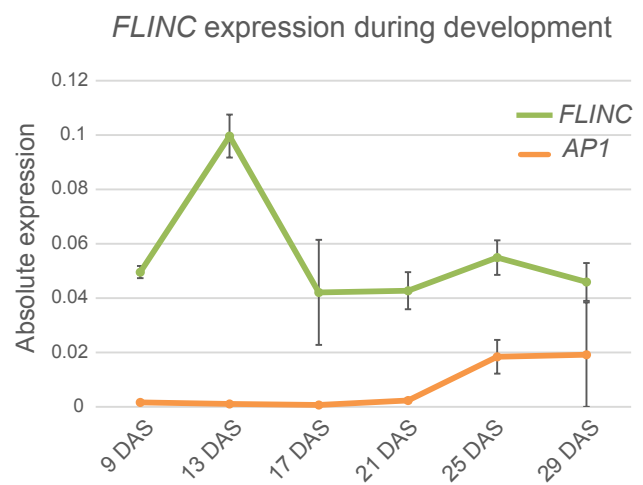

Supplement: Supplementary file 6 — Figure S2. A. FLINC location in the genome. B. FLINC expression in WT and flinc mutant plants. The graph shows the average of three biological replicates, each composed of a pool of 10 2 weeks-old plants. Plants were growing at 21 °C in long day conditions. Bars indicate SEM of the replicates. Plants with a T-DNA insertion in the lncRNA locus do not show detectable expression of the lncRNA transcript. C. At1g56233 expression in WT and flinc mutant plants. The graph shows the average of three biological replicates, each composed of a pool of 10 2 weeks-old plants. Plants were growing at 21 °C in long day conditions. Bars indicate SEM of the replicates. No significant difference in At1g56233 expression was observed in flinc, p-value equals 0.3439 according to the T-test. D. FLINC expression in WT and FLINC-OE plants. A pool of 10 2 weeks-old plants growing on selection medium at 21 °C in long day was used for the analysis. E. FLINC expression measured by qPCR in plants growing at 16 °C and 25 °C in long days. Expression is relative to the level at 16 °C. Bars indicate SEM of two biological replicates, each composed of a pool of seven plants. FLINC expression is significantly lower at 25 °C compared to 16 °C (p-value = 0.0467, Students’ t-test). F. FLINC expression during a 24 h time course in plants grown at 21 °C in long days. The graph shows the average of four biological replicates, each composed of a pool of 25 ten days-old plants. Plants were growing at 21 °C in long day conditions. Bars indicate SEM of the replicates. G. FLINC expression measured by qRT-PCR in different plant tissues. The graph shows the average of three biological replicates, each composed of a pool of 6 to 8 plants for all tissues, except for ‘siliques’ and ‘stems’, for which only two biological replicates were used. Bars indicate SEM between the replicates. Plants were growing at 21 °C in long day conditions. H. FLINC and AP1 expression measured by qPCR in rosettes during a development time c [file 12870_2018_1362_MOESM6_ESM.pdf]

A

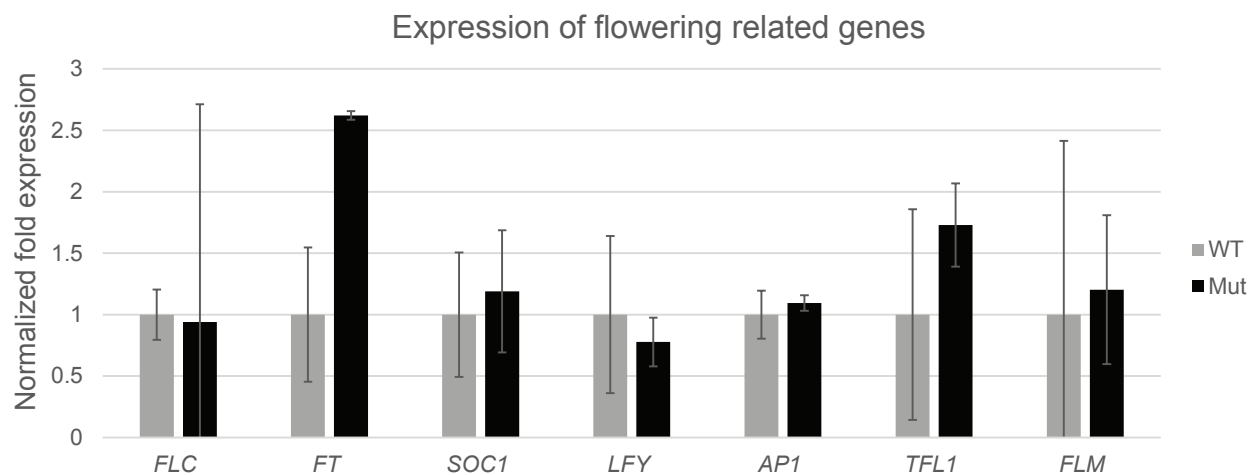

B

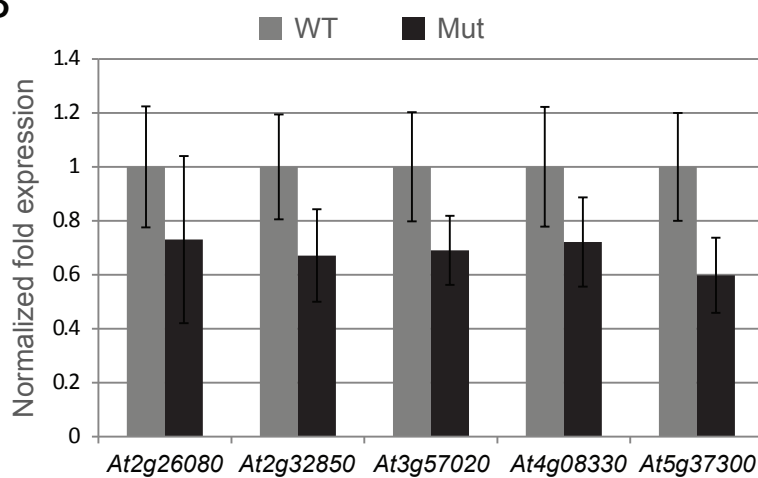

C

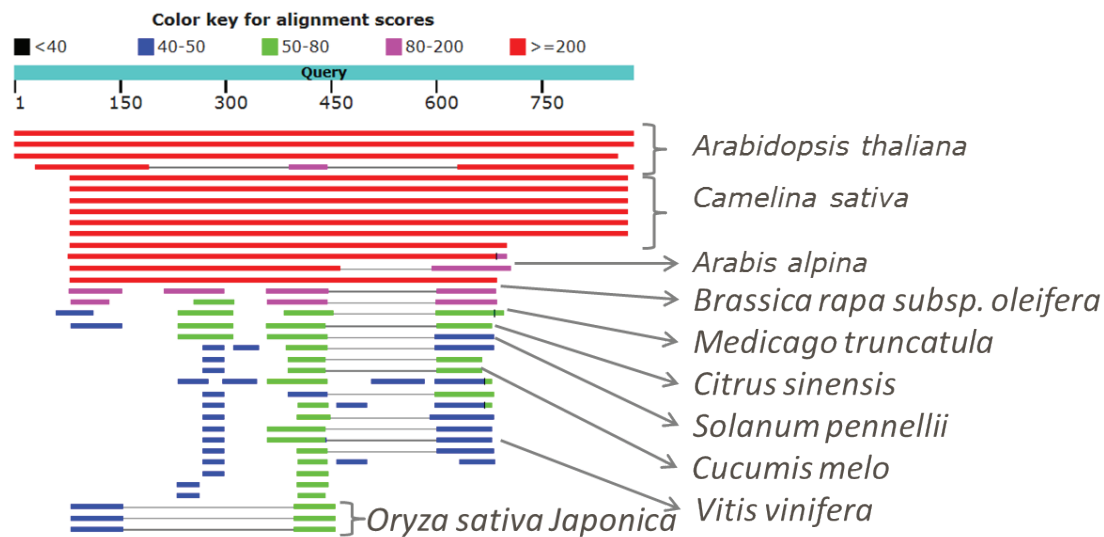

Supplement: Supplementary file 7 — Figure S3. A. Expression of flowering-related genes in WT and flinc plants. Expression was measured by qPCR in rosettes of twenty days-old WT and flinc grown at 16 °C in long day conditions. Material was harvested at ZT6. The graph shows the average of three biological replicates, each composed of a pool of 10 plants. Bars indicate SEM of the replicates. B. Expression of genes with sequence similarity to FLINC in wild-type (WT) and flinc mutant plants (Mut), as measured by qPCR. The graph shows the average of three biological replicates, each composed of a pool of 10 two weeks-old plants growing at 21 °C in long day. Bars indicate SEM of the replicates. C. FLINC sequences are also found in other plant species. (PDF 561 kb) [file 12870_2018_1362_MOESM7_ESM.pdf]
